# Supplementary material for: Delivery of loaded MR1 monomer results in efficient ligand exchange to host MR1 and subsequent MR1T cell activation
Source: Commun Biol. 2024 Feb 24;7:228. doi: 10.1038/s42003-024-05912-4 (PMC10894271; doi:10.1038/s42003-024-05912-4)
Supplement: Supplementary file 3 — Description of Additional Supplementary Files [file 42003_2024_5912_MOESM3_ESM.pdf]

Description of Additional Supplementary Files

File name: Supplementary Data 1

Description: Source data underlying the graphs in the paper.
