# Supplementary material for: Delivery of loaded MR1 monomer results in efficient ligand exchange to host MR1 and subsequent MR1T cell activation
Source: Commun Biol. 2024 Feb 24;7:228. doi: 10.1038/s42003-024-05912-4 (PMC10894271; doi:10.1038/s42003-024-05912-4)
Supplement: Supplementary file 5 — Reporting Summary [file 42003_2024_5912_MOESM5_ESM.pdf]

nature portfolio

Corresponding author(s): Harriff, Melanie

Last updated by author(s): Jan 5, 2024

Reporting Summary

Nature Portfolio wishes to improve the reproducibility of the work that we publish. This form provides structure for consistency and transparency in reporting. For further information on Nature Portfolio policies, see our Editorial Policies and the Editorial Policy Checklist.

Statistics

For all statistical analyses, confirm that the following items are present in the figure legend, table legend, main text, or Methods section.

n/a	Confirmed	
☐	☒	The exact sample size (*n*) for each experimental group/condition, given as a discrete number and unit of measurement
☐	☒	A statement on whether measurements were taken from distinct samples or whether the same sample was measured repeatedly
☐	☒	The statistical test(s) used AND whether they are one- or two-sided<br>*Only common tests should be described solely by name; describe more complex techniques in the Methods section.*
☒	☐	A description of all covariates tested
☒	☐	A description of any assumptions or corrections, such as tests of normality and adjustment for multiple comparisons
☐	☒	A full description of the statistical parameters including central tendency (e.g. means) or other basic estimates (e.g. regression coefficient) AND variation (e.g. standard deviation) or associated estimates of uncertainty (e.g. confidence intervals)
☐	☒	For null hypothesis testing, the test statistic (e.g. *F*, *t*, *r*) with confidence intervals, effect sizes, degrees of freedom and *P* value noted<br>*Give P values as exact values whenever suitable.*
☒	☐	For Bayesian analysis, information on the choice of priors and Markov chain Monte Carlo settings
☒	☐	For hierarchical and complex designs, identification of the appropriate level for tests and full reporting of outcomes
☒	☐	Estimates of effect sizes (e.g. Cohen's *d*, Pearson's *r*), indicating how they were calculated

Our web collection on statistics for biologists contains articles on many of the points above.

Software and code

Policy information about availability of computer code

Data collection	FACS Diva, ELISPOT AID software, Delta Vision acquisition software
Data analysis	FlowJo version 10.6.1 or 10.7.1, Graphpad Prism version 9 or 10, Imaris version 7.3

For manuscripts utilizing custom algorithms or software that are central to the research but not yet described in published literature, software must be made available to editors and reviewers. We strongly encourage code deposition in a community repository (e.g. GitHub). See the Nature Portfolio guidelines for submitting code & software for further information.

Data

Policy information about availability of data

All manuscripts must include a data availability statement. This statement should provide the following information, where applicable:

- Accession codes, unique identifiers, or web links for publicly available datasets
- A description of any restrictions on data availability
- For clinical datasets or third party data, please ensure that the statement adheres to our policy

The authors declare that the data supporting the findings of this study are available within the paper and its supplementary information files.

Research involving human participants, their data, or biological material

Policy information about studies with human participants or human data. See also policy information about sex, gender (identity/presentation), and sexual orientation and race, ethnicity and racism.

Reporting on sex and gender	PBMC from human subjects was only used to make dendritic cells to be used as antigen presenting cells or used to expand T cell clones as described in the methods section. All findings in this manuscript are based on the T cell clone function or on other cell line function, e.g. BEAS-2B cell line. No data is presented on the PBMC from human subject participants and sex and gender were not taken into consideration. PBMC was obtained through IRB approved protocols.
Reporting on race, ethnicity, or other socially relevant groupings	See above
Population characteristics	See above
Recruitment	See above
Ethics oversight	All samples were collected and all experiments were conducted under protocols approved by the institutional review board at Oregon Health and Science University. PBMCs were obtained by apheresis from healthy adult donors with informed consent.

Note that full information on the approval of the study protocol must also be provided in the manuscript.

Field-specific reporting

Please select the one below that is the best fit for your research. If you are not sure, read the appropriate sections before making your selection.

☒ Life sciences ☐ Behavioural & social sciences ☐ Ecological, evolutionary & environmental sciences

For a reference copy of the document with all sections, see nature.com/documents/nr-reporting-summary-flat.pdf

Life sciences study design

All studies must disclose on these points even when the disclosure is negative.

Sample size	Please see above as this study is focused solely on the function of T cell clones or other cell lines. Sample size was not applicable and not considered.
Data exclusions	No data was excluded.
Replication	All data were successfully replicated, the number of times disclosed in each figure legend.
Randomization	This is not applicable, see above.
Blinding	This is not applicable, see above.

Reporting for specific materials, systems and methods

We require information from authors about some types of materials, experimental systems and methods used in many studies. Here, indicate whether each material, system or method listed is relevant to your study. If you are not sure if a list item applies to your research, read the appropriate section before selecting a response.

Materials & experimental systems

n/a	Involved in the study	
☐	☒	Antibodies
☐	☒	Eukaryotic cell lines
☒	☐	Palaeontology and archaeology
☒	☐	Animals and other organisms
☒	☐	Clinical data
☒	☐	Dual use research of concern
☒	☐	Plants

Methods

n/a	Involved in the study	
☒	☐	ChIP-seq
☐	☒	Flow cytometry
☒	☐	MRI-based neuroimaging

Antibodies

Antibodies used	Biotinylated MR1, BioLegend, Biotin Custom Conjugation, 92553, 26.5<br>MR1-APC, BioLegend, 361108, 26.5<br>IgG2a-APC, BioLegend, 400222, MOPC-173<br>MHC-I- APC, BIolegend, 311410, W6/32<br>MR1 unlabeled, OHSU antibody core, 26.5<br>IgG2a unlabeled, BioLegend, 400224, MOPC-173<br>CD3 PerCP BD Biosciences 347344 SK7<br>CD3 BV650 Biolegend 317324 , OKT3<br>CD4 BUV737 BD 612748, SK3<br>CD4 PeCy7 BD Biosciences 348789 SK3<br>CD8 APC Cy7 Biolegend 344714 SK1<br>anti-AP2A1 (BD, 610501)<br>rabbit anti-ACTB (Abcam, ab8227)<br>goat anti-mouse 800CW (LiCor, 926-32210)<br>goat anti-rabbit 680RD (LiCor, 926-68071)<br>anti-human IFN-gamma mAb, Mabtech 3420-3-1000, 1-D1K<br>anti-human IFN-gamma mAb ALP-conjugated, Mabtech 3420-9A-1000, 7-B6-1
Validation	Each primary antibody was titrated using positive target cells. Additionally, isotype or FMO controls were used where necessary to determine nonspecific binding by FcR and to set gates in analysis, respectively.

Eukaryotic cell lines

Policy information about cell lines and Sex and Gender in Research

Cell line source(s)	ATCC (BEAS-2B, THP-1, HEK293T, A549). Human PBMC (Dendritic cells, B-LCL, T cell clones). Thermo Scientific (Hi5, SF9). D426G11 T cell clone, sex male. D481BD1 T cell clone and D481 DC, sex female. D480F6 T cell clone and D480 DC, sex male. D520 DC, sex female.
Authentication	T cell clones are expanded from frozen stocks using published protocols and are used for 2 weeks and then discarded. They are also tested to confirm their phenotype using either ELISPOT or flow cytometry. BEAS-2B cells were bought from ATCC. These cell lines are passaged up to 15 times before being discarded. Freezebacks are frozen down during the first 2-3 passages.
Mycoplasma contamination	All cell lines tested negative for mycoplasma.
Commonly misidentified lines<br>(See ICLAC register)	None

Plants

Seed stocks	Not applicable.
Novel plant genotypes	Not applicable.
Authentication	Not applicable.

Flow Cytometry

Plots

Confirm that:

☒ The axis labels state the marker and fluorochrome used (e.g. CD4-FITC).

☒ The axis scales are clearly visible. Include numbers along axes only for bottom left plot of group (a 'group' is an analysis of identical markers).

☒ All plots are contour plots with outliers or pseudocolor plots.

☒ A numerical value for number of cells or percentage (with statistics) is provided.

Methodology

Sample preparation	This is described in detail for each assay in the methods.
Instrument	BD FACSymphony, LSRII, or Canto cytometer
Software	Acquisition on FACS Diva, Analysis with FlowJo
Cell population abundance	There were no post sort fractions - flow was on either T cell clones or BEAS-2B cell lines.
Gating strategy	For T cell clones: FSC/SSC, live, CD3+, CD4+ or CD8+ depending on clone. For BEAS2B: FSC/SSC, GFP+

☒ Tick this box to confirm that a figure exemplifying the gating strategy is provided in the Supplementary Information.
